# Plasma lipidomics as a diagnostic tool for peroxisomal disorders

**DOI:** 10.1007/s10545-017-0114-7

**Published:** 2017-12-05

**Authors:** Katharina Herzog, Mia L. Pras-Raves, Sacha Ferdinandusse, Martin A. T. Vervaart, Angela C. M. Luyf, Antoine H. C. van Kampen, Ronald J. A. Wanders, Hans R. Waterham, Frédéric M. Vaz

**Affiliations:** 1 Laboratory Genetic Metabolic Diseases, Departments of Clinical Chemistry and Pediatrics, University of Amsterdam, Meibergdreef 9, Amsterdam, 1105 AZ The Netherlands; 2 Department of Clinical Epidemiology, Biostatistics, and Bioinformatics, Academic Medical Center, University of Amsterdam, Meibergdreef 9, Amsterdam, 1105 AZ The Netherlands; 3 Biosystems Data Analysis, Swammerdam Institute for Life Sciences, University of Amsterdam, Science Park 904, Amsterdam, 1098 XH The Netherlands

## Abstract

Peroxisomes are ubiquitous cell organelles that play an important role in lipid metabolism. Accordingly, peroxisomal disorders, including the peroxisome biogenesis disorders and peroxisomal single-enzyme deficiencies, are associated with aberrant lipid metabolism. Lipidomics is an emerging tool for diagnosis, disease-monitoring, identifying lipid biomarkers, and studying the underlying pathophysiology in disorders of lipid metabolism. In this study, we demonstrate the potential of lipidomics for the diagnosis of peroxisomal disorders using plasma samples from patients with different types of peroxisomal disorders. We show that the changes in the plasma profiles of phospholipids, di- and triglycerides, and cholesterol esters correspond with the characteristic metabolite abnormalities that are currently used in the metabolic screening for peroxisomal disorders. The lipidomics approach, however, gives a much more detailed overview of the metabolic changes that occur in the lipidome. Furthermore, we identified novel unique lipid species for specific peroxisomal diseases that are candidate biomarkers. The results presented in this paper show the power of lipidomics approaches to enable the specific diagnosis of different peroxisomal disorders.

**Electronic supplementary material:**

The online version of this article (10.1007/s10545-017-0114-7) contains supplementary material, which is available to authorized users.

## Introduction

Inborn errors of metabolism (IEM) comprise a group of inherited diseases mainly caused by biallelic recessive mutations in genes encoding metabolic enzymes or molecular transporters (Miller et al 2015). The underlying genetic defect of IEM can affect a specific metabolic pathway, including enzymes, transporters or co-factors, or an entire cell organelle. A variety of IEM are associated with impairments in lipid metabolism, and more than 100 different IEM are associated with disturbed lipid synthesis or breakdown (Lamari et al 2014). Lipids are one of the major classes of metabolites, and can be classified into simple lipids, such as fatty acids and sterols, and complex lipids, including phospholipids and sphingolipids (van Meer et al 2008). Phospholipids have a glycerol backbone linked to two fatty acyl chains and a phosphate group, which can be esterified to an amino alcohol. Sphingolipids consist of a sphingoid base, N-acylated fatty acids, and a headgroup consisting of sugars, but also phosphoamino alcohols (van Meer et al 2008). The large variability in structure and functions of lipids is a result of the type of fatty acyl chains, which can differ in chain length, unsaturation, and hydroxylation (Lamari et al 2014). In addition, in complex lipids, different head groups, incorporation of carbohydrates or phosphate residues, and changes in the ether or ester linkage of the backbone to the fatty acyl moiety lead to diversity within the different lipid classes (van Meer et al 2008; Lamari et al 2014).

Lipidomics is an emerging independent branch of metabolomics, aimed to resolve the lipid composition of cells, tissues, and body fluids. In principle, metabolomics approaches allow the discovery and monitoring of disease biomarkers and provide a platform to study pathogenesis and progression of disease (Houten 2009). Whereas an increasing number of novel genetic variants leading to IEM are identified by next-generation sequencing, metabolomics is a promising tool to study disease pathophysiology (Lamari et al 2014; Miller et al 2015). Furthermore, lipidomics approaches are employed to study changes in lipid profiles in disease pathogenesis and to identify lipid biomarkers for diagnosis and monitoring of disease (Vaz et al 2014).

In this study, we demonstrate the potential of lipidomics using plasma samples from patients with peroxisomal disorders. In this group of IEM, the biogenesis of peroxisomes is impaired due to genetic mutations in one of 14 *PEX* genes impacting multiple peroxisome-depending metabolic pathways (peroxisome biogenesis disorders, PBDs), or one peroxisomal metabolic pathway is affected due to a defective enzyme or membrane transporter (peroxisomal single-enzyme deficiencies, PEDs) (Waterham et al 2016). Peroxisomes are crucial cell organelles for lipid metabolism, involved in both catabolic and anabolic processes, such as the α- and β-oxidation of various fatty acids, and the biosynthesis of plasmalogens and bile acids (Wanders et al 2010). Together, peroxisomal disorders affect one in 5000 newborns (Waterham et al 2016).

The PBDs are divided into the Zellweger spectrum disorders (ZSDs), rhizomelic chondrodysplasia punctata type 1 and 5 (RCDP, OMIM #215100), and peroxisomal fission defects (Steinberg et al 2006; Waterham et al 2016). The ZSDs comprise a disease spectrum, ranging from severe, lethal clinical presentations to milder, late-onset progressive forms of the disease. Common clinical parameters are hypotonia, liver dysfunction, sensorineural hearing loss, and ocular abnormalities (Steinberg et al 2006). ZSDs are caused by mutations in any of 13 *PEX* genes, leading to defects in peroxisome assembly or in import of peroxisomal matrix proteins (Waterham and Ebberink 2012). In RCDP, mutations in the *PEX7* gene (type 1), which codes for the PTS2- protein receptor, or in the *PEX5* gene affecting the isoform PEX5L, which acts as a co-receptor in the PTS2-import pathway (type 5), result in defective import of several matrix proteins involved in plasmalogen biosynthesis and α-oxidation (Waterham et al 2016). Clinically, RCDP is characterized by congenital cataracts, skeletal deformations, craniofacial dysmorphism, proximal shortening of the limbs, and stippled calcifications of cartilage tissue (Waterham and Ebberink 2012).

In PEDs, one of the peroxisomal enzymes or membrane proteins is affected, which often leads to specific biochemical phenotypes as a result of the impaired metabolic function. D-bifunctional protein (DBP) deficiency (OMIM #261515) catalyzes the second and third step of peroxisomal β-oxidation, accepting both straight- and branched-chain fatty acids as substrate (Ferdinandusse et al 2006). DBP deficiency is caused by mutations in the *HSD17B4* gene, and the clinical presentation markedly resembles that of patients with a ZSD and ACOX1 deficiency (Ferdinandusse et al 2006; Waterham et al 2016). The enzyme α-methylacyl-CoA racemase (AMACR) is involved in the isomerization of fatty acids containing a 2-methyl group from the (R)-configuration to the corresponding (S)-configuration (Ferdinandusse et al 2000). This conversion allows these fatty acids, including pristanic acid and the C27-bile acid intermediates, to be substrates for peroxisomal β-oxidation (Waterham et al 2016). Deficiency of AMACR (OMIM #614307) results in liver failure in neonates, and peripheral neuropathy, epilepsy, retinitis pigmentosa, and intermittent encephalopathy in adult patients (Ferdinandusse et al 2000; Waterham et al 2016). Refsum disease (OMIM #266500) is caused by mutations in the *PHYH* gene encoding phytanoyl-CoA 2-hydroxylase, which is a crucial enzyme in peroxisomal α-oxidation (Wanders et al 2011). This pathway converts 3-methyl fatty acids, such as phytanic acid, into 2-methyl fatty acids, which are further degraded by peroxisomal β-oxidation. Refsum disease patients often present at a later stage of life, and clinical symptoms include progressive retinitis pigmentosa, deafness, anosmia, and polyneuropathy (Waterham et al 2016).

In this paper, we present lipidomics as a novel approach to study peroxisomal disorders. By performing a lipidomics analysis using ultra-high performance liquid chromatography coupled to high-resolution mass spectrometry (UPLC-HRMS), we show in this study that the lipid composition is altered in plasma samples from patients with a peroxisomal disorder. The changes in the profiles of phospholipids, di- and triglycerides, and cholesterol esters correspond with the characteristic metabolite abnormalities that are currently used in the metabolic screening for these diseases. The lipidomics approach, however, gives a much more detailed picture of the metabolic changes that occur in the lipidome, and also reflect the heterogeneity of peroxisomal disorders.

## Results

We used a lipidomics approach to investigate the lipid composition of plasma samples from patients with peroxisomal disorders, including RCDP, ZSD, DBP and AMACR deficiency, and Refsum disease. We measured the samples using normal and reversed-phase chromatography in positive and negative ionization mode, and annotated 1365 distinct phospholipids, cholesterol esters, and di- and triglyceride species (Suppl. Table A).

**Fig. 1:**
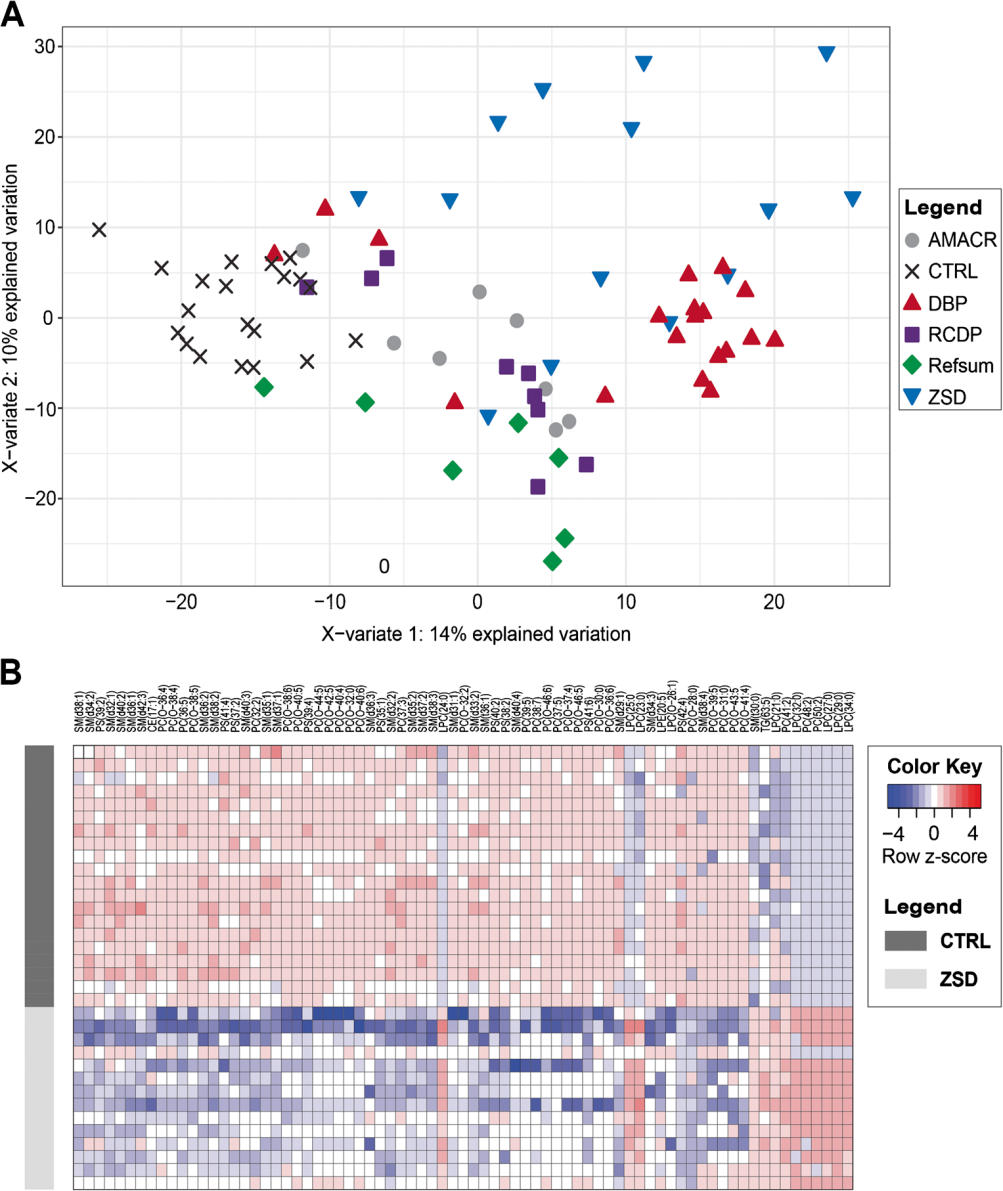
Phospholipid profiles in plasma from patients with peroxisomal disorders. **a**) Score plot of the multivariate model based on PLS-DA depicting variations in phospholipid profiles. Shown are the first (X-variate) and second component (Y-variate) of the model, the percentage of variance explained by the components is indicated in parentheses. **b**) Un-supervised hierarchical clustering plot of phospholipid species with the highest VIP score as determined by OPLS-DA between plasma samples from ZSD patients and controls. Data were logarithm-transformed, and colors in the heat-map reflect the logarithm of the relative metabolite abundance (z-score): red color indicates higher, and blue color indicates lower values than the mean abundance per metabolite

Using partial least squares regression discriminant analysis (PLS-DA), we investigated the clustering pattern of all samples based on the lipid profiles (Worley and Powers 2013). In the PLS-DA score plot, the first component (X-variate 1) explains the largest variability in the data. Most DBP and ZSD plasma samples were clustered and clearly separated from the control group, indicating large differences in lipid composition, whereas samples from the other groups were clustered in-between the DBP, ZSD, and control samples (Fig. 1a). In addition, a few patient samples clustered close to the control group, indicating a lipid profile more similar to the controls. On the second component (X-variate 2), plasma samples from patients with Refsum disease and ZSD were separated from the control group, clustering in different directions on the X-variate (Fig. 1a).

## Disease-specific lipid profiles in PBD plasma samples

Since PLS-DA did not result in a clear separation of all groups, and the quality assessment statistic (Q^2^ = 0.109) was low, we used another supervised discriminant analysis technique to compare each disease group to the control group separately. We performed orthogonal projections to latent structures discriminant analysis (OPLS-DA), which achieved good separation between the different disease groups and the control group in all comparisons (Suppl. Fig. 1). We extracted the variable importance in projection (VIP) scores to rank all annotated lipid species based on their contribution to the separation of the groups.

For plasma samples from ZSD patients, phosphatidylcholine (PC) species containing >40 carbon atoms in combined side chains (including very long-chain fatty acids, VLCFAs), and Lyso PC (LPC) containing VLCFAs (≥ 22 carbon atoms) were increased when compared to plasma samples from control individuals (Fig. 1b). A variety of PC ether phospholipid species and sphingomyelin species with 33 to 40 carbon atoms in combined side chains were decreased in ZSD patient plasmas. In addition, a number of PC species with an uneven chain number or polyunsaturated fatty acyl chains (PUFAs) were decreased in these samples when compared to plasma samples from control individuals (Fig. 1b). In summary, these data show marked changes in the lipid composition, most prominently the accumulation of phospholipid species containing VLCFAs, and decreased levels of several PC ether phospholipids.

In plasma samples from patients with RCDP, various ether phospholipid species of PC, LPC, and phosphatidylethanolamine (PE) with different summed fatty acyl chain length were decreased when compared to plasma samples from control individuals (Suppl. Fig. 1).

## DBP phenotype correlates with lipid profiles

In plasma samples from patients with DBP deficiency, various PC and LPC species containing VLCFAs were increased when compared to plasma samples from control individuals, similar to ZSD plasma samples. PC ether phospholipids containing PUFAs with 38 to 44 carbon atoms in combined side chains were decreased in plasma samples from patients with DBP deficiency, as well as a number of PC and phosphatidylserine (PS) species (Suppl. Fig. 2). These results indicate that, like for ZSD, phospholipid species containing VLCFAs accumulate in plasma from DBP-deficient patients, whereas species containing PUFAs were decreased.

**Fig. 2:**
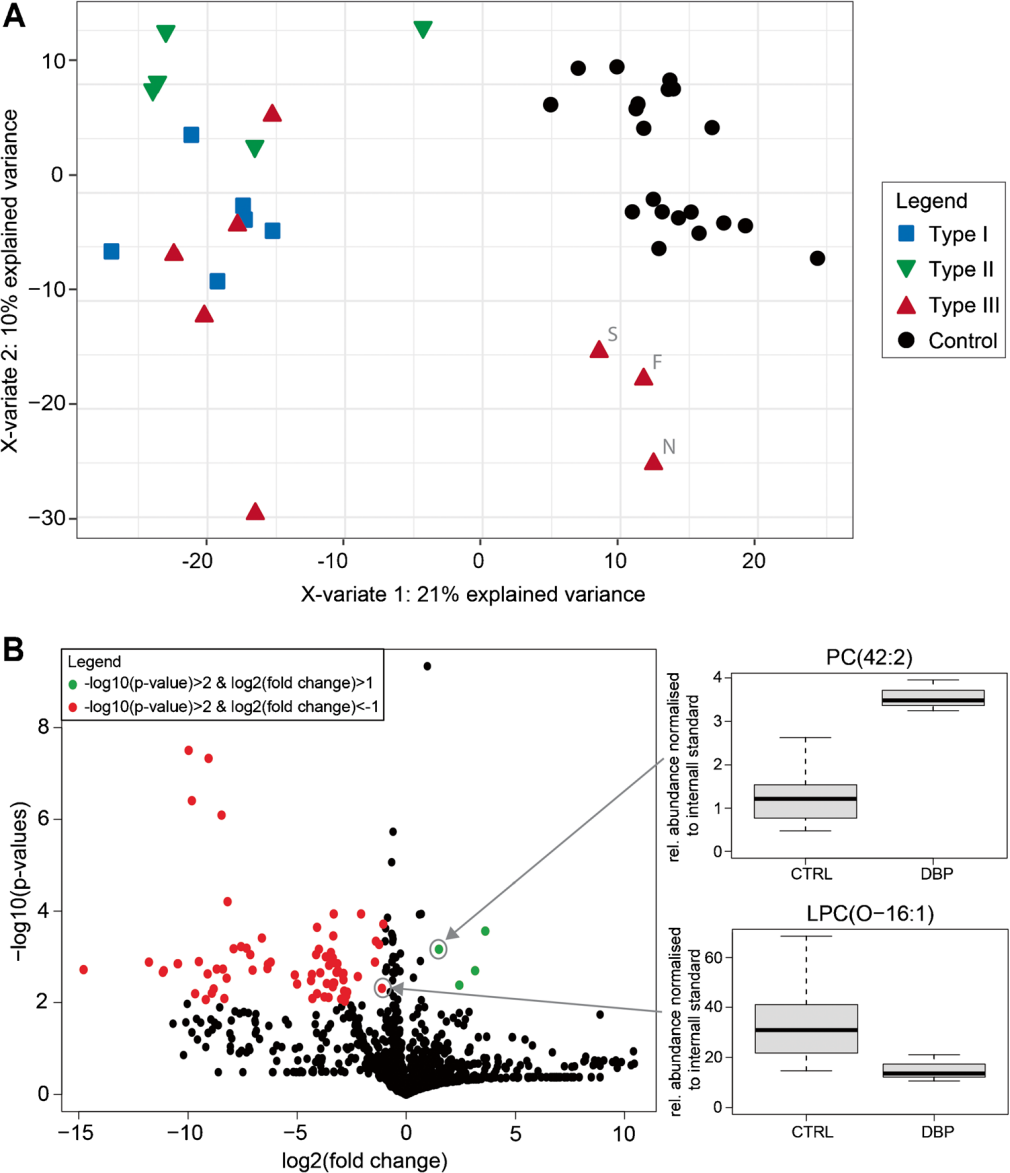
Differences in phospholipid composition in plasma samples from patients with DBP deficiency. **a**) Score plot of the multivariate PLS-DA model of plasma samples from patients with DBP deficiency classified by disease severity. The first (X-variate) and second component (Y-variate) of the PLS-DA model are depicted, and the percentage of variance explained by the components is indicated in parentheses. Plasma samples from three very mild type III DBP patients are highlighted (sample no. F, N, and S). **b**) Left panel: volcano plot of plasma samples from three very mild type III DBP patients as indicated in (a) versus samples from controls. Phospholipid species that were increased in plasma samples from DBP patients when compared to controls are depicted as green dots, and species that were decreased in plasma samples from DBP patients are depicted as red dots. Right panel: two examples of statistically significantly different phospholipid species as shown in the left panel are shown as box-and-whisker plots

Despite the good separation of the control and patient group in the OPLS-DA, the plasma samples from DBP patients showed differences in the phospholipid composition within the group, as represented in the score plot, whereas the plasma samples from control individuals nicely clustered together (Fig. 2a). It is known that the disease severity of DBP deficiency ranges from severe to relatively mild phenotypes. A complete deficiency of both the hydratase and dehydrogenase activity of DBP is associated with a severe phenotype (type I), whereas isolated deficiencies of either the hydratase (type II) or dehydrogenase activity (type III) can display a more attenuated phenotype (Ferdinandusse et al 2006). Since a number of samples from DBP-deficient patients clustered close to the control samples (Fig. 2a), we investigated whether these had a milder phenotype. We classified the different patients with DBP deficiency as described previously based on survival (Ferdinandusse et al 2006), and could show that the three samples with a relatively mild phenotype based on prolonged survival clustered in close proximity to the control group in the PLS-DA score plot (Fig. 2a). Next, we identified lipid species that were significantly different in these three plasma samples when compared to plasma samples from healthy individuals (*p*-value <0.01, absolute log2 fold change >1). Mainly LPC, PC, and PS species were significantly changed in plasma samples from mild DBP-deficient patients, such as LPC(O-16:1) and PC(42:2) (Fig. 2b, Suppl. Table B). Although the levels of some phospholipid species were very low in plasma samples from patients with DBP deficiency, all these species were discriminative between the DBP and the control group, making them potentially good diagnostic markers.

## Discovery of unique phospholipid species containing branched-chain fatty acids

In plasma samples from patients with AMACR deficiency, PS species with PUFAs containing 37 to 39 carbon atoms in combined side chains were decreased when compared to plasma samples from control individuals (Suppl. Fig. 1). In addition, PC and PC ether phospholipid species with PUFAs were relatively decreased. Triglyceride (TG) species with 59 to 69 carbon atoms in combined side chains, diglyceride (DG) species, as well as a number of PC and LPE ether phospholipid species were increased in AMACR-deficient patient plasma samples when compared to plasma from control individuals (Suppl. Fig. 2). These data indicate changes in lipid composition, especially of lipid species with uneven fatty acyl chain length and species containing PUFAs.

In plasma samples from patients with Refsum disease, a variety of PS, PC, and LPC were increased when compared to plasma samples from healthy individuals (Supp. Fig. 1). Notably, several of these species were hydroxylated phospholipids. In addition, a number of PC ether phospholipid and PS species likely containing a VLCFA and a PUFA were decreased in plasma samples from patients with Refsum disease when compared to samples from control individuals.

**Fig. 3:**
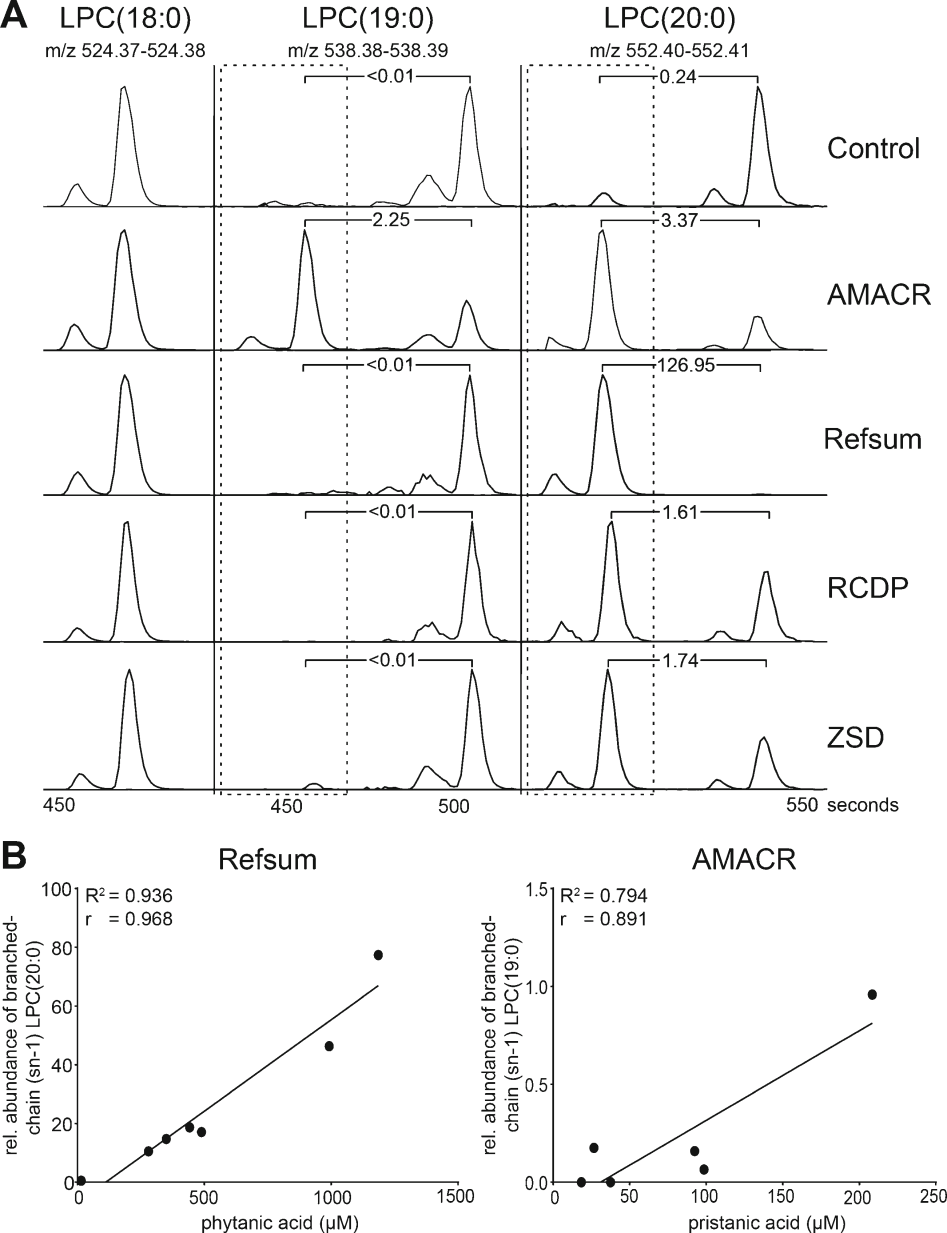
Unique phospholipid species in plasma from patients with Refsum disease and AMACR deficiency. **a**) Representative extracted ion chromatograms of straight-chain and branched-chain fatty acids (highlighted by dotted line). The ratios of the branched-chain peak (sn-1)/straight-chain peak (sn-1) are indicated. **b**) Scatter plot of the correlation of phytanic acid and branched-chain (sn-1) LPC20:0 levels in plasma samples from patients with Refsum disease (left panel), and pristanic acid and branched-chain (sn-1) LPC(19:0) levels in plasma samples from patients with AMACR deficiency (right panel) are shown. The coefficient of determination (R^2^) indicates the estimate of goodness of fit of the linear regression model, and the Pearson correlation coefficient (r) indicates the measure of correlation

Based on the lipid species that were elevated in plasma samples from patients with AMACR deficiency, RCDP type 1 and 5, and Refsum disease, we assumed that branched-chain fatty acids are incorporated into phospholipid species, as the accumulation of the branched-chain fatty acids pristanic acid (2,6,10,14-tetramethylpentadecanoic acid) and phytanic acid (3,7,11,15-tetramethylhexadecanoic acid) are biochemical hallmarks in patients with AMACR deficiency, and RCDP and Refsum disease, respectively (Wanders et al 2010; Ferdinandusse et al 2016). For instance, PC(42:2) and DG(39:3) were increased in AMACR deficiency, and LPC(20:0) and PC(38:0) were increased in Refsum disease patients (Suppl. Fig. 2). To find evidence for this, we focused on species that were measured with reversed-phase chromatography followed by MS analysis in the positive ion mode. With this experimental setup, isobaric species of different structural isomers (e.g., with different fatty acid composition) can be distinguished by small differences in retention time (RT). The saturated branched-chain fatty acids pristanic and phytanic acid both have four methyl groups, and therefore are chemically distinct from their corresponding straight-chain fatty acids. Since lysophospholipids only have one fatty acyl chain, we focused on LPC species and selected saturated LPC species with a fold-change >10 in the diseased versus the control group. In plasma samples from patients with AMACR deficiency, the typical extracted ion chromatogram showed four peaks for LPC(19:0), of which the peaks with lower RT were highly abundant in this disease group, likely corresponding to pristanoyl-LPC (Fig. 3a). For both straight- and branched-chain LPC(19:0), the fatty acid can be linked to the glycerol backbone at the sn-1 or sn-2 position, resulting in two separate peaks. Pristanoyl-LPC(19:0) levels showed a trend to correlate with total pristanic acid levels as determined by GC-MS. In plasma samples from Refsum disease patients, four peaks for LPC(20:0) were present in the average ion chromatogram, of which the peaks with lower RT were highly abundant, likely corresponding to phytanoyl-LPC (Fig. 3a). Notably, the same species were also observed in plasma samples from patients with AMACR deficiency, RCDP type 1 and 5, and ZSD patients (Fig. 3a). Phytanoyl-LPC(20:0) levels correlated well with total phytanic acid levels as determined by GC-MS (Fig. 3b).

## Lipidomics data correlate with known peroxisomal biomarkers

**Fig. 4:**
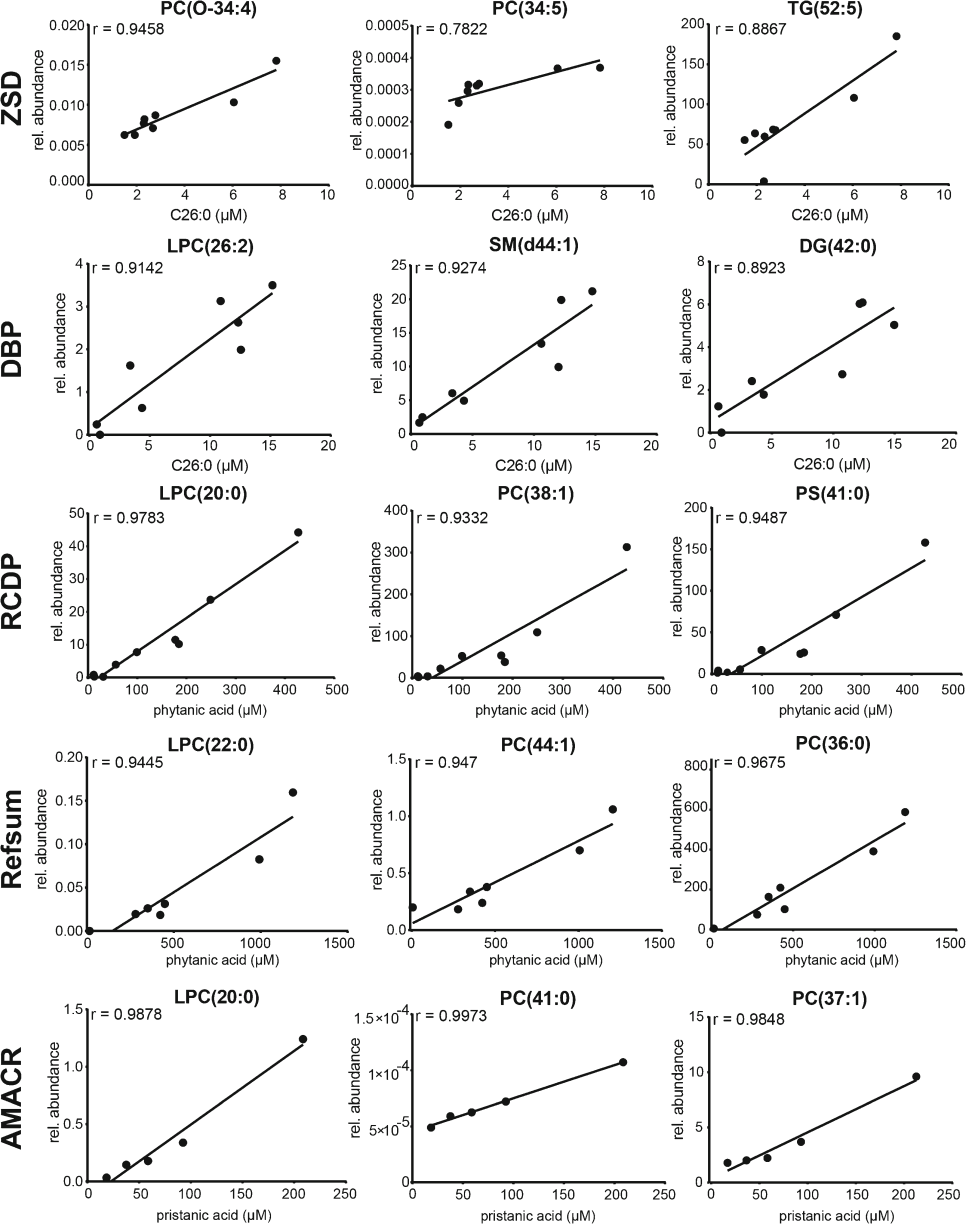
Correlation of phospholipid species with aberrant metabolites used for diagnosis of peroxisomal disorders. Scatter plots showing the correlations of (phospho)lipid species levels (as indicated for each plot) and total C26:0 levels in plasma samples from ZSD patients, and patients with DBP deficiency; total phytanic acid levels in plasma samples from patients with RCDP and Refsum disease; and total pristanic acid levels in plasma samples from patients with AMACR deficiency. Pearson correlation coefficient (r) indicates the measure of correlation

We investigated the correlation of the measured (phospho)lipid species determined by lipidomics with the metabolite abnormalities determined by GC-MS, which are routinely used for diagnosis of peroxisomal disorders. We compared VLCFA levels, i.e., the total C26:0 levels, as determined during diagnostic work-up, with the (phospho)lipid levels obtained by lipidomics in the same plasma samples from patients with a ZSD or DBP deficiency. We found good correlations of C26:0 levels with the levels of a number of phospholipid species, such as PC(O-34:4) in plasma samples from ZSD patients, and LPC(26:2) in plasma samples from patients with DBP deficiency (Fig. 4). The finding of a positive correlation of VLCFA levels with increasing levels of what likely represents an ether phospholipid is unexpected, but the exact structure of PC(O-34:4) remains to be established. In addition, we compared the total phytanic acid levels with the lipidomics data in plasma from patients with Refsum disease and RCDP type 1 and 5, and total pristanic acid levels with the (phospho)lipid levels obtained in plasma samples from patients with AMACR deficiency. We found good correlations of both phytanic levels and pristanic acid levels with the levels of a variety of phospholipid species in plasma samples from Refsum disease, RCDP type 1 and 5, and AMACR deficiency, respectively (Fig. 4).

## Materials and methods

### Plasma samples

Samples were collected from residual diagnostic plasma samples in our laboratory, and were anonymized for this study. All blood samples were initially preserved in ethylenediaminetetraacetic acid (EDTA), and plasma was isolated prior to storage at −20 °C. We used plasma samples from eight patients with AMACR deficiency, 19 patients with DBP deficiency, nine patients with RCDP type 1 or 5, six patients with Refsum disease, and 14 patients with a ZSD. Patient samples were selected based on clinical diagnoses, which were confirmed previously by biochemical testing and molecular genetic analyses. For controls, we used 20 plasma samples from ten anonymized healthy adult individuals, and ten patients where the metabolic screen, including full peroxisomal screening, was negative.

### Biochemical analyses, phospholipid extraction procedure and UPLC-HRMS

Lipidomic analysis was performed as described previously (Herzog et al 2016). In short, two UPLC separations, using a reversed phase and a normal phase, were analyzed in the positive and negative ion mode resulting in four data files per sample. VLCFAs, phytanic acid, and pristanic acid levels were measured as described previously (Vreken et al 1998). For correlation analysis, only results from diagnostic work-up determined in the same plasma samples were used.

### Bioinformatics and statistics

The lipidomics data were processed using an in-house developed metabolomics pipeline (Herzog et al 2016) using the R package “xcms” (Smith et al 2006). Data in figures are presented as mean ± SD. Statistical analysis was performed using student’s t-test or one-way ANOVA with post-hoc correction according to the method of Benjamini and Hochberg (q-values). Partial least squares regression discriminant analysis (PLS-DA), and orthogonal projections to latent structures discriminant analysis (OPLS-DA) with the extraction of variable importance in projection (VIP) scores was performed using the R packages “ggplot2”, “mixOMICS”, and “ropls” (Thévenot et al 2015).

## Discussion

Peroxisomes are important organelles for cellular lipid metabolism, and a defect in peroxisome function results in multi-systemic diseases with specific clinical and biochemical disturbances that originate from the affected peroxisomal pathways (Waterham et al 2016). In this paper, we performed lipidomics using UPLC-HRMS to study the lipid composition in plasma samples from patients with a peroxisomal disorder affecting either peroxisome biogenesis, including ZSD and RCDP type 1 and 5, or one specific peroxisomal pathway, including Refsum disease, DBP and AMACR deficiency. The focus of this initial study was to explore whether lipidomic profiles in plasma can be used to detect metabolic disorders that affect lipid metabolism, without aiming to comprehensively characterize the disease lipidome itself. We found characteristic changes in the composition of phospholipids, di- and triglycerides, and cholesterol esters, which reflect the heterogeneity of peroxisomal disorders that depends on their specific biochemical profiles.

The plasma samples we used in this study were collected during routine care and therefore were not matched by gender, age, or method of collection (fasted/fed, etc.), and the samples had been stored for different time periods. Despite this heterogeneity, the changes in phospholipid profiles we found in the plasma samples are comparable to the changes in phospholipid composition that have previously been reported in fibroblasts and other tissues from patients with peroxisomal disorders (Pettus et al 2004; Abe et al 2014; Dorninger et al 2015; Herzog et al 2016, 2017; Yagita et al 2017), suggesting that the plasma lipidome reflects the changes in tissues. This also indicates that the pre-analytical conditions do not negatively influence the lipidomic detection of marked changes in plasma phospholipid profiles and that lipidomic characterization is likely applicable to diagnose peroxisomal disorders.

The phospholipid composition in plasma samples from patients with peroxisomal disorders reflects the biochemical abnormalities found in metabolic tests that are currently used in diagnostic screening for peroxisomal disorders. These include elevated levels of VLCFAs in plasmas from patients with ZSD and DBP deficiency, decreased levels of ether phospholipids, including plasmalogens, in plasmas from patients with ZSD and RCDP type 1 and 5, and elevated levels of phytanic acid and pristanic acid in Refsum disease and AMACR deficiency, respectively (Waterham et al 2016). We found various (phospho)lipid species that correlated well with the levels of the classical biomarkers for peroxisomal disorders.

Plasmalogen measurement for diagnostic purposes is exclusively performed in erythrocytes (Ferdinandusse et al 2016), as only small amounts of plasmalogens can be detected in plasma samples using gas chromatography (Moser et al 1999). Interestingly, however, in this study we show that we can readily detect a variety of ether phospholipids, including plasmalogens, in plasma samples using a lipidomics approach, and that they are deficient in ZSD, RCDP, and DBP deficiency. Aberrant ether phospholipid levels were also found in fibroblasts from patients with PEDs, including DBP deficiency (Herzog et al 2017) but also have been reported in neurological disorders such as Alzheimer’s disease (Berger et al 2016). The exact mechanism behind decreased ether phospholipid levels in plasma from DBP-deficient patients should be further investigated. Our results demonstrate that the lipidomic findings in plasma can be used as part of the diagnostic approach to screen for disorders where plasmalogens are deficient, and possibly obviates the need for a separate erythrocyte plasmalogen measurement. Yet, this has to be validated in a larger cohort of patients by direct comparison of the two methods.

In Refsum disease, phytanic acid accumulates due to a defect in peroxisomal α-oxidation (Wanders et al 2010). Similar to the incorporation of VLCFAs into phospholipids, we observed that phytanic acid is most likely incorporated into specific phospholipid species. Recently, phytanic and pristanic acid were shown to be converted into phytanoyl- and pristanoyl-carnitine in plasma samples from patients with Refsum disease and AMACR deficiency, respectively (Herzog et al 2017). In the current study, we identified a number of phospholipid species in plasma samples from Refsum disease patients that most likely contain phytanic acid. Similarly, we detected various species containing uneven chain length in AMACR deficiency, likely representing lipid species containing pristanic acid. These species correlated well with the total levels of phytanic acid, and to a lesser extent with pristanic acid, respectively. These characteristic phospholipid species could be novel diagnostic biomarkers when lipidomics is used as a screening tool for peroxisomal diseases such as ZSDs, Refsum disease, RCDP, and AMACR deficiency. Surprisingly, many hydroxylated phospholipid species were found to be elevated specifically in Refsum disease. A possible explanation for this could be that the extremely high phytanic acid levels in combination with enhanced ω-oxidation in this disorder (Wierzbicki 2007; Wanders et al 2010) yields hydroxylated branched fatty acids (intermediates of the ω-oxidation) that are also incorporated in complex lipids.

Using our lipidomics platform, only 9125 (7%) features were assigned (of which 1365 lipid species were uniquely annotated), whereas the majority of the 138,622 measured peak groups is yet unknown. Further studies are needed to elucidate the identity of other candidate features, which may potentially be used as novel biomarkers. This study shows that a broad lipidomic analysis may result in unexpected, but highly interesting findings (i.e., the positive correlation of PC(O-34:4) with VLCFA levels). Further analysis of the exact structure of the metabolites, the underlying biological mechanisms, and diagnostic relevance of these findings will be a subject of future studies.

In conclusion, we performed lipidomics using UPLC-HRMS and showed that the lipidome was clearly altered in plasma samples from patients with different peroxisomal disorders. The characteristic changes in phospholipid profiles were in line with the biochemical hallmarks of the disorders that are currently analyzed by several different metabolite analyses. We also identified novel lipid species for specific peroxisomal diseases that are candidate biomarkers, which all were measured in the same analytical run. By further optimizing this approach we aim to develop a method that enables the specific diagnosis of the different peroxisomal disorders using a single plasma lipidomic analysis.

## Electronic supplementary material

ESM 1 (DOCX 7396 kb)

ESM 2 (XLSX 9555 kb)

ESM 3 (XLSX 36 kb)

## Abbreviations

AMACR: α-methylacyl-CoA racemase
DBP: D-bifunctional protein
DG: diglyceride
IEM: inborn errors of metabolism
LPC: lyso-phosphatidylcholine
LPE: lyso- phosphatidylethanolamine
OPLS-DA: orthogonal projections to latent structures discriminant analysis
PBD: peroxisome biogenesis disorder
PC: phosphatidylcholine
PE: phosphatidylethanolamine
PED: single peroxisomal enzyme deficiency
PLS-DA: partial least squares regression discriminant analysis
PS: phosphatidylserine
PUFA: poly-unsaturated fatty acyl chain
RCDP: rhizomelic chondrodysplasia punctata type 1 and 5; RT, retention time
TG: triglyceride
UPLC-HRMS: ultra-high performance liquid chromatography coupled to high-resolution mass spectrometry
VIP: variable importance in projection
VLCFA: very long-chain fatty acid
ZSDs: Zellweger spectrum disorders
