# Supplementary material for: Plasma lipidomics as a diagnostic tool for peroxisomal disorders
Source: J Inherit Metab Dis. 2017 Dec 5;41(3):489–98. doi: 10.1007/s10545-017-0114-7 (PMC5959966; doi:10.1007/s10545-017-0114-7)
Supplement: Supplementary file 1 — (DOCX 7396 kb) [file 10545_2017_114_MOESM1_ESM.docx]

**Supplemental Files**

**Supplemental Figure 1.**


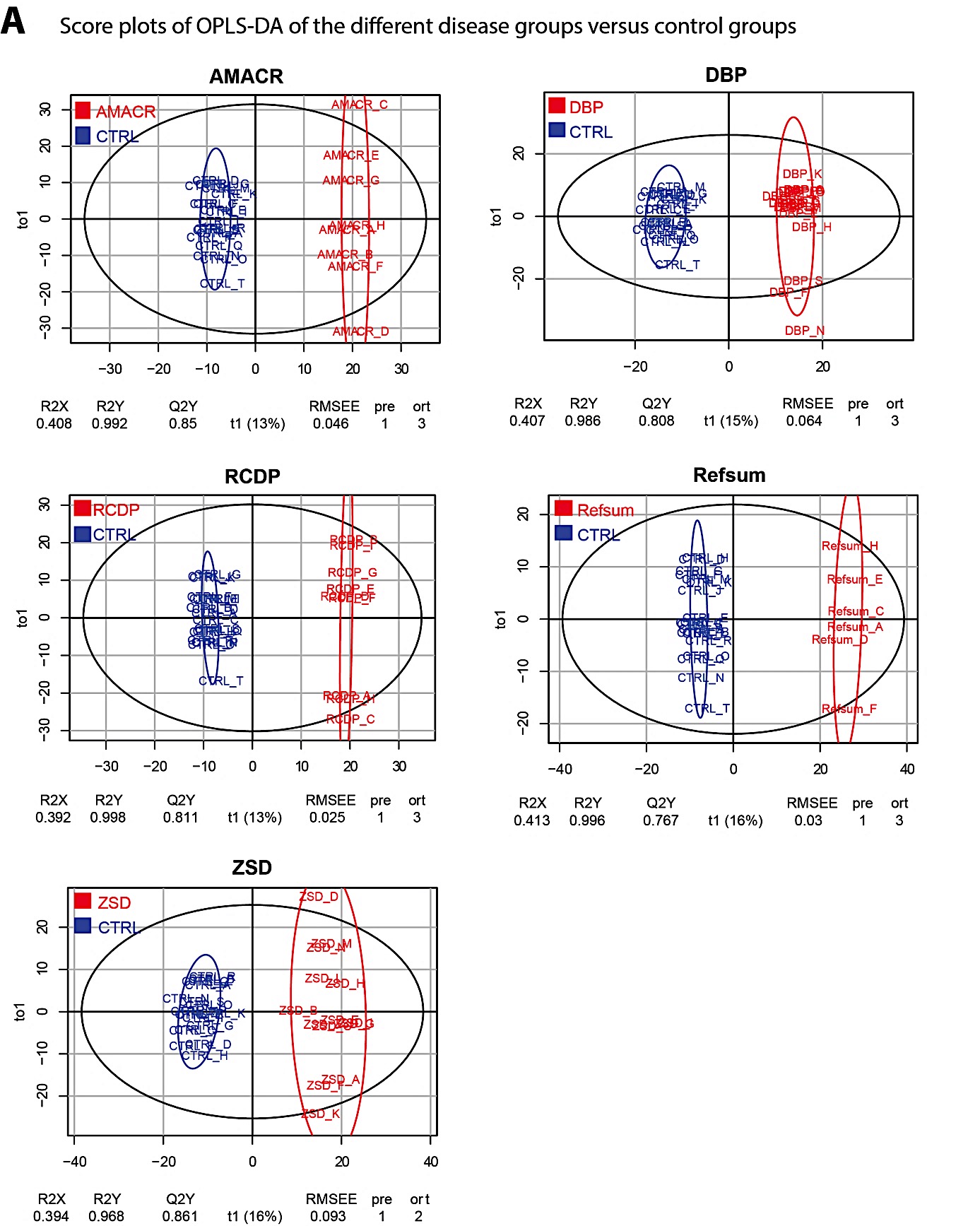


Score plot of the OPLS-DA models of variations in phospholipid composition for the different disease groups versus the control group as indicated. Ellipses around each group represent the 95% confidence regions of the group. The percentage of variance explained in the predictor component (t1) is indicated in parentheses. The percentage of predictor variance explained by the full model (RX2), the percentage of response variance (RY2), and the predictive performance of the model estimated by cross-validation are indicated.

**
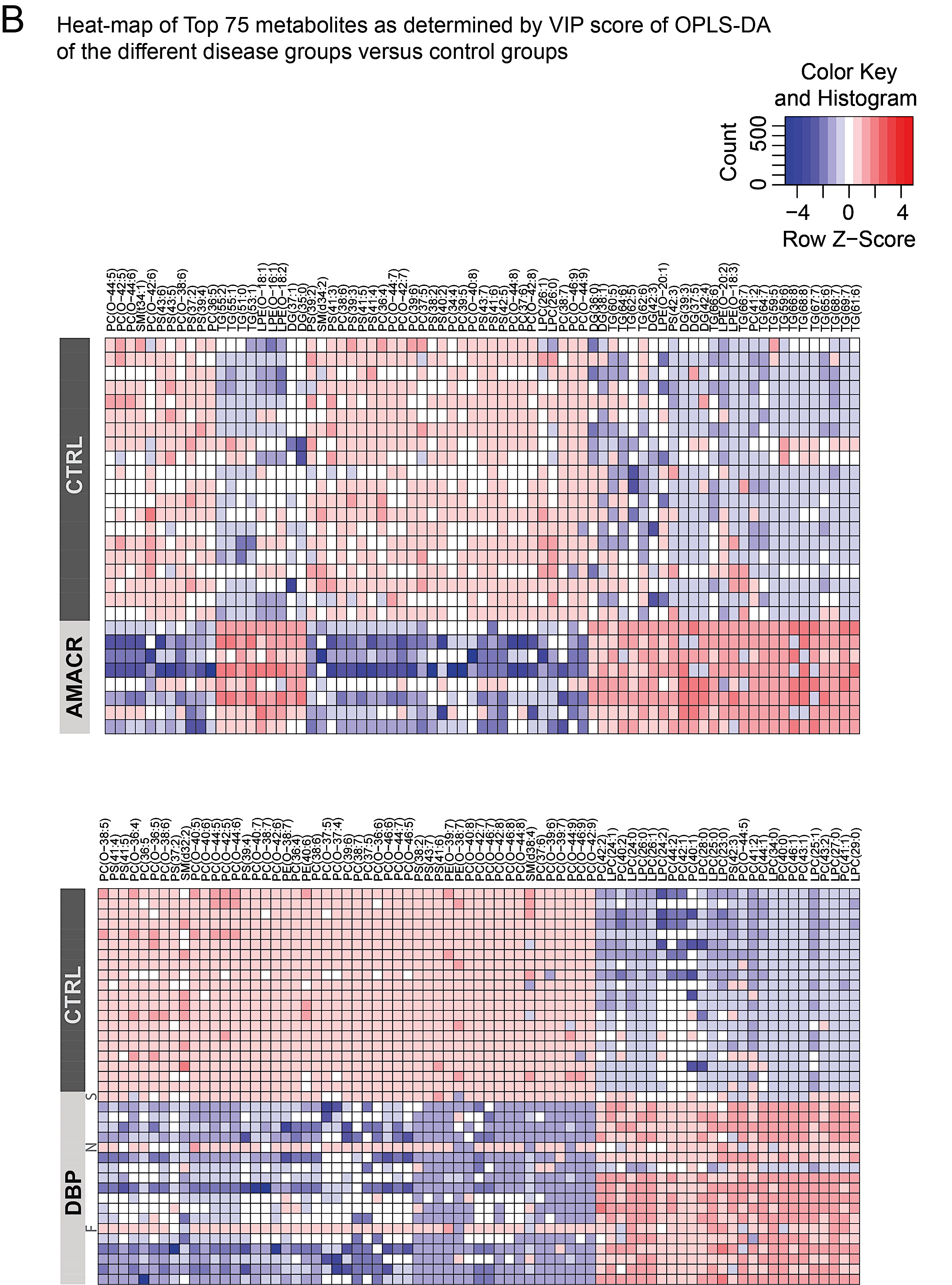
Supplemental Figure 2.**

**A**


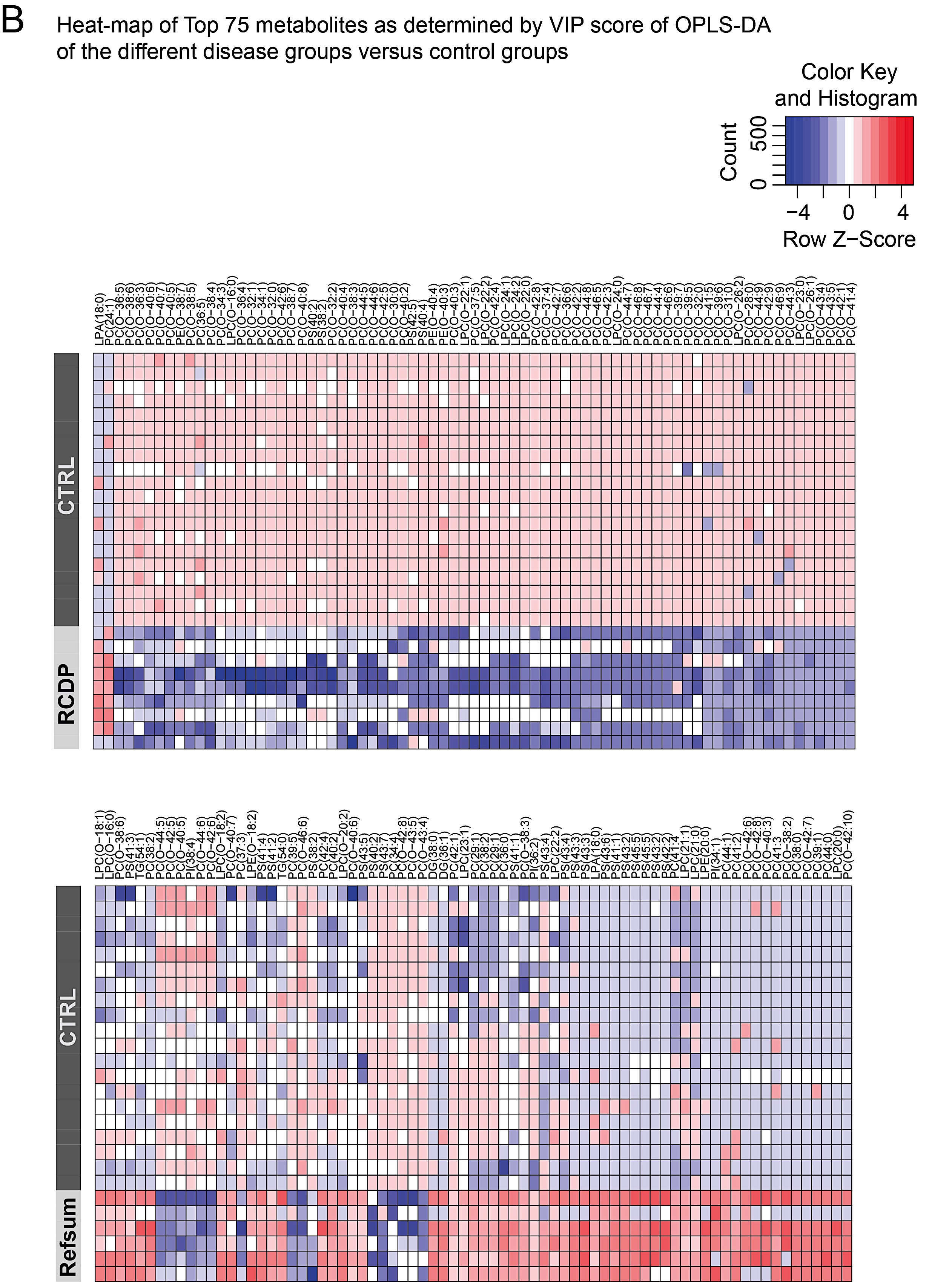
**B**

Un-supervised hierarchical clustering plot of phospholipid species with the highest VIP score as determined by OPLS-DA between plasma samples per disease group and controls. A) Upper panel: AMACR deficiency, lower panel: DBP deficiency. Plasma samples from three very mild type III DBP patients as indicated in Figure 2 are highlighted (sample no. F, N, and S). B) Upper panel: RCDP, lower panel: Refsum disease. Data were logarithm-transformed, and colours in the heat-map reflect the logarithm of the relative metabolite abundance (z-score): red colour indicates higher, and blue colour indicates lower values than the mean abundance per metabolite.
